# Antimicrobial use surveillance in broiler chicken flocks in Canada, 2013-2015

**DOI:** 10.1371/journal.pone.0179384

**Published:** 2017-06-28

**Authors:** Agnes Agunos, David F. Léger, Carolee A. Carson, Sheryl P. Gow, Angelina Bosman, Rebecca J. Irwin, Richard J. Reid-Smith

**Affiliations:** 1 Public Health Agency of Canada, Center for Foodborne, Environmental and Zoonotic Infectious Diseases, Guelph, Ontario, Canada; 2 Public Health Agency of Canada, Center for Foodborne, Environmental and Zoonotic Infectious Diseases, Saskatoon, Saskatchewan, Canada; Wageningen Universiteit en Researchcentrum IMARES, NETHERLANDS

## Abstract

There is a paucity of data on the reason for and the quantity of antimicrobials used in broiler chickens in Canada. To address this, the Canadian Integrated Program for Antimicrobial Resistance Surveillance (CIPARS) implemented surveillance of antimicrobial use (AMU) and antimicrobial resistance (AMR) in broiler chicken flocks in 2013. Shortly after this (2014), the poultry industry banned the preventive use of ceftiofur in broiler chickens. The objectives of this analysis were to describe antimicrobial use (AMU) in Canadian broiler chickens between 2013 and 2015 (n = 378 flocks), compare these results to other animal species in Canada, to highlight the utility of farm surveillance data to evaluate the impact of a policy change, and to explore how different antimicrobial use metrics might affect data interpretation and communication. The surveillance data indicated that the poultry industry policy resulted in lower antimicrobial use and resistance, and they successfully captured information on when, where, why, and how much antimicrobials were being used. The majority of antimicrobials were administered via the feed (95%). The relative frequency of antimicrobial classes used in broiler chickens differed from those used in swine or in food animal production in general. Coccidiostats were the most frequently used antimicrobial classes (53% of total kg). Excluding coccidiostats, the top three most frequently used antimicrobial classes were bacitracin (53% of flocks), virginiamycin (25%) and avilamycin (21%), mainly used for the prevention of necrotic enteritis. Depending on the AMU metric utilized, the relative rankings of the top antimicrobials changed; hence the choice of the AMU metric is an important consideration for any AMU reporting. When using milligrams/Population Correction Unit (mg/PCU) the top three antimicrobial classes used were bacitracins (76 mg/PCU), trimethoprim-sulfonamides (24 mg/PCU), and penicillins (15 mg/PCU), whereas when using a number of Defined Daily Doses in animals using Canadian standards /1,000 chicken-days at risk (nDDDvetCA/1,000 CD) the ranking was bacitracins (223 nDDDvetCA/1,000 CD), streptogramins (118 nDDDvetCA/1,000 CD), and trimethoprim-sulfonamides (87 nDDDvetCA/1,000 CD). The median animal treatment days in feed for one cycle (ATD/cycle) during the three-year study were 34 ATD/cycle; this was equal to the mean age of the flocks at pre-harvest sampling day (days at risk), indicating that the studied flocks except those that were raised without antibiotics and organic, were fed with medicated rations throughout the observation period. Overall, more than half (59%) of antimicrobials used in broiler chickens were in classes not used in human medicine, such as ionophores and chemical coccidiostats aimed to prevent coccidiosis. Compared to grower-finisher pigs and in production animal species (national sales data), the mg/PCU of antimicrobials used in broiler chickens was relatively lower. The findings of this paper highlighted the importance of farm-level AMU surveillance in measuring the impact of interventions to reduce antimicrobials in poultry.

## Introduction

The World Health Organization’s (WHO’s) Global Action Plan on Antimicrobial Resistance (AMR) included recommendations for the monitoring of antimicrobial use (AMU) through surveillance and research to help mitigate the dissemination and emergence of AMR organisms in both animals and humans [1]. In 2015, Canada developed a federal action plan on AMR with three main areas of focus: surveillance, stewardship and innovation [2]. Currently, the activities of the Public Health Agency of Canada’s (PHAC) Canadian Integrated Program for Antimicrobial Resistance Surveillance (CIPARS) align with both the federal action plan and with the strategic objectives described in the WHO’s Global Action Plan and the OIE Terrestrial Animal Code [3]. AMU surveillance activities in animals and humans provide context to understand AMR arising from the food chain, and are important for measuring trends over time, for making comparisons between animal species, for AMR risk assessment and for benchmarking [4].

Global consensus on animal AMU data collection and reporting methods do not yet exist, but many activities to achieve this are underway. In Europe, several member countries of the European Union (EU) and European Economic Area routinely report the total amount of antimicrobial sold in food animals as milligrams of active ingredient, adjusted by animal populations and weights [Population Correction Unit (PCU)]. The data are reported on an annual basis to the European Medicines Agency’s European Surveillance for Veterinary Antimicrobial Consumption (ESVAC) [5]. In March 2017, the European Medicines Agency published the draft ‘Guidance on Provision of Data on Antimicrobial Use by Animal Species from National Data Collection Systems’ for a 6-month public consultation period [6]. This guidance document described herd/flock-level national surveillance framework designs (census and sample survey options) for the collection of AMU in European member countries. European Medicines Agency’s Defined Daily Doses in animals (DDDvet) and Defined Course Doses in animals (DCDvet) standards were also developed by ESVAC to provide guidance to European member countries for tracking AMU over time by animal species, while accounting for average drug dose [7,8]. The AMU indicators DDDvet, DCDvet and mg/PCU were described in the draft guidance [6].

Broiler chicken specific AMU studies have also described surveillance approaches, explored various AMU metrics, discussed the attributes of these metrics, and have shown how data are being used to inform prudent use practices in the poultry industry [9–12]. In Canada, antimicrobial products are approved for the prevention and treatment of commonly diagnosed bacterial and protozoal diseases of broiler chickens. However, little is known about the reasons for, the frequency of, and the overall quantity of antimicrobials used at the hatchery or the farm. To address this, CIPARS, in collaboration with FoodNet Canada (PHAC’s sentinel site based food-borne disease surveillance program) and the poultry industry, developed a surveillance framework for AMU and AMR data collection in broilers and turkeys, similar to existing CIPARS swine AMU/AMR farm-based data collection [13]. Surveillance was initiated in 2013. The timing was opportune as the surveillance started just prior to the broiler industry implementation of their AMU reduction initiatives outlined in their industry sector-wide strategy [14]. Also, as Canada transitions to the removal of growth promotion and/or production claims of medically-important antimicrobial drugs and enhanced veterinary oversight of AMU in food animals [15,16], the farm-level CIPARS data, including the poultry data, will provide a reference point for animal AMU.

With the global and national importance of collecting AMU data and the previous gap in knowledge of use of antimicrobials in broilers in Canada, the objectives of this analysis were to describe temporal changes in AMU from 2013 to 2015 from sentinel broiler chicken farms in Canada, to compare various AMU metrics or indicators, to compare the relative distribution of AMU classes in broiler chickens, grower-finisher pigs and national antimicrobial distribution data, and to determine if the CIPARS data would detect the effects of a use reduction policy in the broiler industry.

## Materials and methods

### Antimicrobial use data sources

Currently all AMU data collection in Canada is voluntary. The broiler chicken farm component of CIPARS was implemented in April 2013 in five provinces (British Columbia, Alberta, Saskatchewan, Ontario and Québec). The number of flocks surveyed each year ranged from 99 to 143 flocks based on the framework described elsewhere [13] and summarized in S1 Table. Briefly, CIPARS allocated the number of flocks proportional to the broiler production profile of the poultry-producing province/region. Only one flock per farm was visited per year. Each flock, defined as a group of birds, hatched and placed in a single production unit such as barn, floor or pen approximately the same day, was assigned a unique flock code. A farm pertains to a registered premise/establishment and may have multiple barns. Only coded farm information was provided to CIPARS to maintain the anonymity of the producer. The participating sentinel veterinarians, who represented approximately 90% of the poultry veterinary practices in Canada, selected the farms based on their practice profile and specific inclusion and exclusion criteria. Farms included were Safe, Safer, Safest^™^ (the on-farm food safety assurance program for broiler chickens in Canada) compliant quota-holding broiler operations. Antibiotic-free, raised without antibiotics or organic production systems were selected proportional to the veterinarian’s practice profile. Veterinarians ensured that selected farms were representative of all the Canadian Hatcheries Federation member hatcheries supplying chicks, representative of the feed mills supplying feeds in the province of their practice, and were geographically distributed (i.e., represented all administrative districts within the province/region and not neighboring flocks). Additionally, these farms were demographically reflective of the veterinary practice and overall broiler industry profile (e.g., variety of flock management: poor to excellent performing flocks, variety in volume of chicks placed: low to high flock densities). These criteria helped ensure that the flocks enrolled were representative of most broiler flocks raised in Canada. To account for seasonal variations of pathogen prevalence and AMU, veterinarians were also asked to distribute their sampling visits across the year.

On these farms, questionnaires were used to collect AMU data (described in detail elsewhere [16]). Briefly, for feed medications, diet (or ration)-specific information was obtained, including the total days each ration was fed, the concentration(s) of active ingredient(s) in the feed, the primary reason(s) for that AMU and the main disease syndromes/pathogens targeted by the drug. For water administration, the data collected were similar to those in feed, and included active ingredient(s), dosage (per liter of drinking water), duration of water treatment, the proportion of flock exposed, the reason(s) for use, and whether the product was prescribed by a veterinarian or was an over the counter purchase. Hatchery-level AMU included the drug name, the final dose per hatching egg or chick, the proportion of chicks or hatching eggs medicated and the reason for use.

Antimicrobial distribution data (2013–2015) obtained from the Canadian Animal Health Institute (CAHI) and AMU data from the swine farm component of CIPARS (2013–2015) were used to compare the relative frequency of use of different antimicrobial classes and quantitative estimates of total use over the last three years. The antimicrobial distribution data were collected by CAHI through a network of data providers (pharmaceutical manufacturers and distributors). CIPARS AMU data collection on grower-finisher swine farms was implemented in 2006; sampling method and questionnaire were similar to the broiler farm program. Swine farm AMU data collection and the CAHI data methods and reporting are described in detail elsewhere [13].

### Estimation of total antimicrobial use, average treatment days

**Fig 1:**
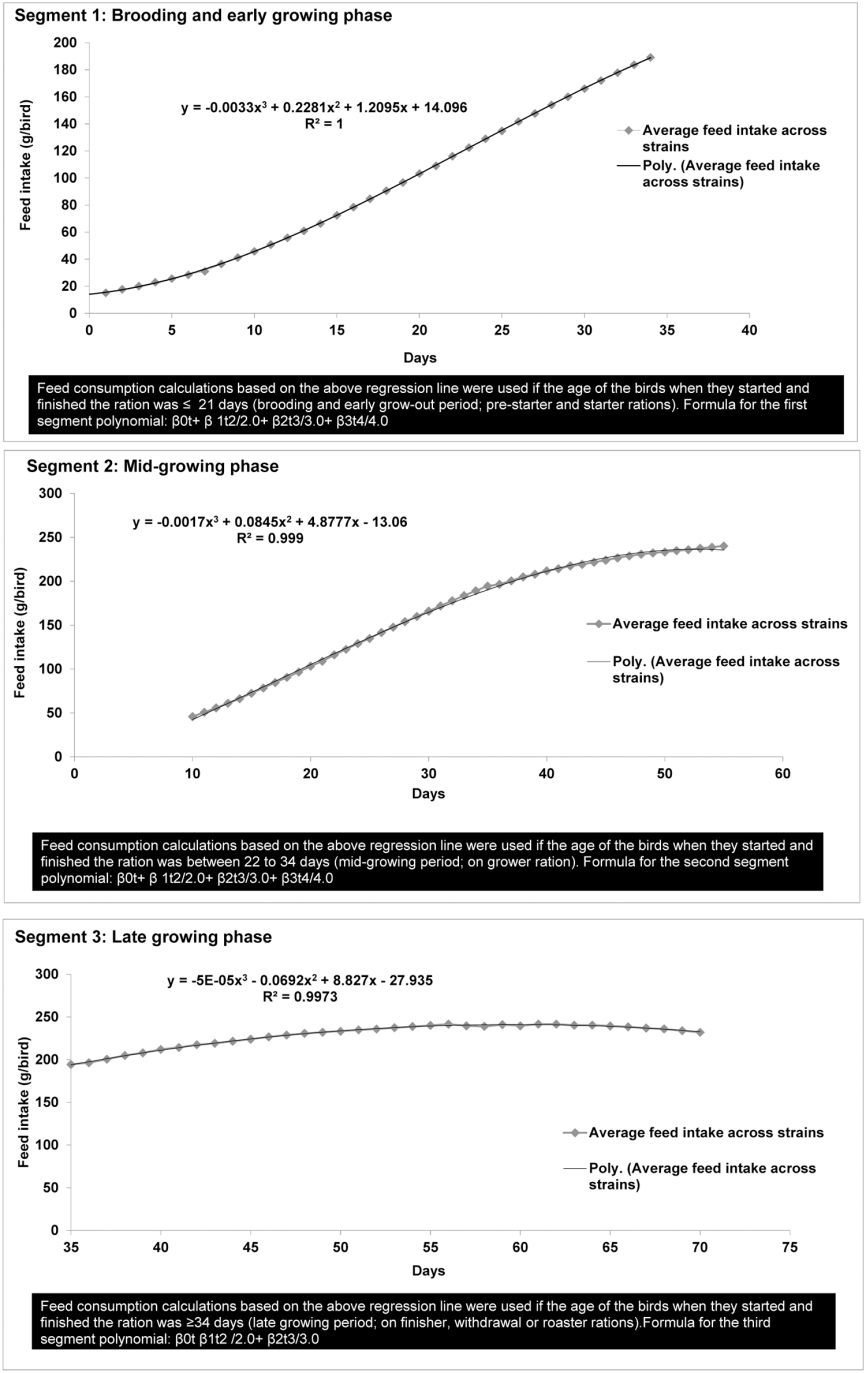
Segments one to three of the daily feed intake (g/day) based on common broiler chicken breeds and Canadian feeding guidelines.

Estimates of feed intake per ration were based on simple regression and integral calculus using feed consumption standards for common breeds raised in Canada (Ross 308 and 708, Cobb 500 and 700) and standards developed by Canadian feed companies (Nutreco Canada Inc.-Shur Gain, Wallenstein Feed and Supply Ltd.) for as-hatched broilers (i.e., males and females combined). CIPARS used this estimation approach because the initial data regarding tonnage fed was more reflective of the tonnage delivered, rather than necessarily consumed by the flock. Yearly updates to the standards were necessary to reflect the year-specific broiler efficiency targets (e.g., feed intake, water intake, weights). From these standards, the cumulative feed consumption was calculated using the average of all feeding standards for broilers and a plot of daily feed consumption in grams per bird. Detailed calculations have been previously described [13]. Briefly, the start and end age of the birds for each ration (e.g., pre-starter, starter, grower, and finisher) was entered in the database. Since the last day of one ration is the start day of the next, an algorithm was used to prevent overlapping days for each subsequent ration. Regression parameters were calculated within Microsoft Excel (Office 14) by using the plotted feed intake curve. A minimum R-square value of > 0.99 was required to be considered a good fit; therefore, to obtain the best fitting regression values, the feeding curve was divided into 3 segments (Fig 1) to estimate the consumption. From the regression coefficients, feed consumption could then be calculated using integral calculus. The area under the curve for each regression equation provided an estimate of feed consumption. The feed consumed was converted to tonnes fed and then multiplied to the level of drug in the product. The total feed derived from the regression equation was used in Eq 1 below to estimate the total milligrams of active ingredient in feed. The total broiler population below is the sum of all broiler flocks for one grow-out cycle only, minus the reported mortality rate at the time of the visit.

Equation 1. Total milligrams in feed

$$\left(Total\;broilers\right)\times feed\;\left(kg\right)\times level\;of\;drug\left(\frac{mg\;drug}{kg\;feed}\right)=Mg_{feed}$$ (1)

For water medications, an approach using three regression lines similar to the above method for feed estimation was applied (S1 Fig), in which the daily water consumption chart from Nutreco Canada, Inc.-Shur-Gain was utilized [13]. The total liters derived from the equation were used in the formula below. The level of drug pertains to the reported inclusion rate in the water (mg/L), accounting for the drug concentration in the product.

Equation 2. Total milligrams in water

$$\left(Total\;broilers\right)\times water\;consumption\;\left(L\right)\times level\;of\;drug\;in\;the\;water\;\left(\frac{mg}{Liter}\right)=Mg_{water}$$ (2)

For injectable antimicrobials, the total mg of active ingredient was calculated by multiplying the number of chicks medicated by the final mg of active ingredient injected per hatching egg/chick.

Equation 3. Total milligrams injected

$$\left(Total\;broilers\right)\;\times \;mg\;per\;hatching\;egg\;or\;chick=Mg_{injection}$$ (3)

The quantity of antimicrobials for each of the three routes of administration was summed to generate the overall total kilograms of active ingredient used in the following estimates.

Equation 4. Population Correction Unit (PCU). The biomass, or Population Correction Unit (PCU) pertains to the total number of birds surveyed (equivalent to one grow-out cycle; population minus half the mortality rate as in Eqs 1–3 above) multiplied by the ESVAC standard weight at treatment for a broiler chicken (1 kg) [5]. One PCU is equivalent to 1 kg broiler chicken.

(Total broilers, 1 cycle) × 1 kg =PCU (4)

Equation 5. mg/PCU–total milligrams consumed via all routes of administration adjusted for broiler population (one broiler grow-out cycle) and weight.

Antimicrobials in feed (mg) + water (mg)+injection (mg)PCU(kg)= mgPCU (5)

Equation 6. nDDDvetCA–number of Defined Daily Doses in animals using Canadian standards.

#### Development of Canadian DDDvet standards

The average labelled dose for each antimicrobial based on Canadian drug product inserts (DDDvetCA_mg_) was assigned following similar methodology to ESVAC’s DDDvet assignment [8]. The average labelled dose was determined as follows: each antimicrobial was assigned a DDDvetCA_mg_ by obtaining all approved unique doses (prevention and treatment purposes) from two Canadian references [17,18] or from expert opinion where no labeled product existed [extra-label drug use (ELDU)], and then the sum of all the doses was divided by the total number of unique doses. The DDDvetCA standards used in this paper are summarized in S2 Table. Because the labeled dose varied by pharmaceutical form (e.g., g/tonne for products administered via feed, g/L water for products administered via the drinking water, mg/chick or hatching eggs for injectable products), values were standardized in mg_drug_/kg_animal_ based on the ESVAC approach [8]. As with the ESVAC methodology, for combination products, the DDDvetCA_mg_ for each antimicrobial component was determined. The ESVAC broiler standard weight at treatment was 1 kg, thus, no further calculation was done to obtain the DDDvetCA values per broiler chicken.

#### Application of the standard to the use data

Subsequently, for each antimicrobial, the assigned DDDvetCA_mg_ was used to adjust the quantity consumed in mg to obtain the number of DDDvetCA.

$$\frac{Antimicrobials\;in\;feed\;\left(mg\right)+water\;\left(mg\right)+injection\;\left(mg\right)}{nDDDvetCA_{mg}}=\text{nDDDvetCA}$$ (6)

Equation 7. nDDDvetCA/1,000 chicken-days at risk (CD). The final step was adjustment of the nDDDvetCA for population (Total broilers in Eqs 1–3) and mean number of days each for one production cycle for the monitored flocks (year-specific days at risk: 2013 and 2014: 34 days: 2015: 35 days). This equation calculates the nDDDvetCA/1,000 chicken-days per antimicrobial agent based on previously described method, treatment incidence [12] with necessary modifications (i.e., the year-specific days at risk was used).

Total antimicrobials (mg)/DDDvetCAmg(Total broilers1,000 ×Days at risk)= nDDDvetCA1,000 CD (7)

Equation 8. Animal treatment days/cycle (ATD/cycle). Animal treatment days/cycle, a metric which is independent of biomass, kilograms or antimicrobial potency, was based on the method described elsewhere [10]. The method was modified to account for the data being only collected for one broiler grow-out cycle. Per broiler flock, the ATD/cycle was determined using the equation below. The mean number of days (days at risk) in this equation was the same number used in Eq 7 above. The equation calculates the treatment days in feed only as the duration of treatments via water and injection at day of hatch were relatively shorter, and oftentimes coincided with the feeding duration of at least one medicated ration.

$$\frac{Flock\;population\;\times no.\;treatment\;days,\;flock\;specific}{Flock\;population\;\times totaldays\;in\;the\;cycle,\;flock\;specific}\;\times Days\;at\;risk\;=\text{ATD/cycle}$$ (8)

### Data analysis- frequency of use and temporal analysis

Responses to questionnaires were entered into a PostGreSQL database and data were extracted in Microsoft Excel (Office 14) for descriptive analysis using either SAS 12.1 (Cary, North Carolina) or Stata 14 (StataCorp, College Station, Texas). The frequency of use for each antimicrobial administered from incubation/hatch to end of growth was summarized by route of administration (*in ovo* or subcutaneous, water, feed), reasons for use, specific disease/syndrome treated, and season (summer, fall, winter). For temporal analyses, the most recent surveillance year (2015, referent year) was compared to the initial surveillance year (2013) and previous year (2014) using logistic regression models (asymptotic or exact models depending on prevalence of the outcome variable). Models were developed with year as a categorical independent variable and using *P* ≤0.05 for significance. Variation in seasonality of use was also assessed; summer (May to August growing period; referent season) was compared to fall (September to December) and winter (January to April). One season represented at least two quota periods. A quota period in the Canadian broiler allocation calendar is equivalent to 8 production weeks (S1 Text) in Canada’s supply management system [19].

Trends in quantity of antimicrobials (kg, mg/PCU, nDDDvetCA/1,000 CD) by class were analyzed descriptively using SAS 12.1, Stata 14 or Microsoft Excel. Changes between 2013 and 2014 and between 2014 and 2015 are simply referred to as percent change in quantity [i.e., more recent surveillance year (2015) minus the previous year (2014), divided by the previous year (2014), then multiplied by 100].

The ATD/cycle was analyzed descriptively using Stata 14; the same software was used for creating the histograms and density plots (kdensity option).

### Data limitations

The CIPARS farm surveillance was designed to collect information from one broiler grow-out cycle or one grow-finishing period for pigs per year. Data from one flock cycle cannot be extrapolated to six cycles (i.e., average turn of the barn in Canadian broiler farms per year) because of seasonal variations in antimicrobial use (e.g., rotational use of antimicrobials for treating necrotic enteritis and coccidiosis as described in the results section), broiler quota allocations (e.g., variation in total birds raised per cycle) and other health and operational factors that may impact antimicrobial use.

## Results

### Farm characteristics

The annual number of flocks varied from 99 to 143 per year. The increase in the number of flocks in 2014 to 2015 was due to additional flocks in Ontario and Alberta (i.e., FoodNet Canada sentinel sites) and the expansion of the surveillance program to other provinces (e.g., Saskatchewan). The total number of flocks surveyed over the three years was 378 flocks. The flocks surveyed represented a total of 2.3 to 3.2 million birds per year (Table 1) and a total of 8.6 million birds over the three-year period. The broiler population, or denominator used in mg/PCU and nDDDvetCA/1,000 CD calculations, represented the sum of all birds for one grow-out cycle. The chicks placed on these farms were mainly Ross and Cobb strains supplied by all the major commercial broiler hatcheries (n = 19) in the provinces/regions that were included in surveillance. The mean age at end of the grow cycle (pre-harvest) was 34 days and birds averaged 2.00 kg live weight.

**Table 1:** Quantity of antimicrobials used (kg) and broiler populations in the CIPARS sentinel flocks, 2013–2015. Data are from one grow-out cycle per flock was monitored.

	2013	2014	2015	Period total(kg)
	(kg)	(kg)	(kg)	
**Route of administration**				
*Including coccidiostats and other*^#^				
Feed	819	1,125	1,053	2,997
Water	15	81	46	142
Injection	0.3	0.4	1	1
Total	819	1,125	1,053	2,997
*Excluding coccidiostats and other*^§^				
Feed	311	424	403	1,138
Water	14	79	43	135
Injection	0	0	1	1
Total	311	424	403	1,138
**Broiler population (number of broiler birds)**^¥^				
	2,298,639	3,297,028	3,035,442	8,631,108

^#^Estimates included ionophores, chemical coccidiostats and flavophospholipids.

^§^Estimates excluded the ionophores, chemical coccidiostats and flavophospholipids. These estimates were used in other quantitative metrics shown in Fig 2.

^¥^Broiler population at chick placement minus half of the mortality rate; this is the summation of all broiler flocks surveyed.

### Frequency of drug use and reasons for use

Tables 2–4 summarize the number of flocks reporting AMU by route of administration.

**Table 2:** Period summary (2013–2015) of antimicrobial use via feed in broiler chicken flocks, frequency, reasons for use and seasonal variations.

		2013	2014	2015^¥^	Total	Reasons for use, period total				Seasonal variations ^§^		
		n = 97	n = 141	n = 135	n (%)	Treatment	Prevention	GP	Diseases targeted	Summer	Fall	Winter
	**Antimicrobials**											
II	Penicillin G potassium	0	5	5	10 (3%)	0%	2%	0%	Necrotic enteritis	5%	3%	0%
	Penicillin G procaine	12	12	13	37 (10%)	1%	9%	1%	Necrotic enteritis	3%	**12%↑**	10%
	Trimethoprim-sulfadiazine	15	17	15	47 (13%)	12%	0%	0%	Neonatal diseases, airsacculitis	10%	14%	10%
II	Tylosin	7	28	20	55 (15%)	0%	15%	0%	Necrotic enteritis	**16%**	10%	**35%↑**
	Virginiamycin	45	**28**	**22↓**	95 (25%)	0%	23%	2%	Necrotic enteritis	29%	23%	29%
III	Bacitracin	47	82	69	198 (53%)	0%	49%	3%	Necrotic enteritis	56%	53%	50%
	Oxytetracycline	1	1	2	4 (1%)	1%	0%	0%	Necrotic enteritis	0%	2%	0%
IV	Bambermycin	1	0	7	8 (2%)	0%	0%	2%	n/a	0%	3%	0%
N/A	Avilamycin	0	**33**	**46↑**	79 (21%)	0%	21%	0%	Necrotic enteritis	**8%**	**29%↑**	6%
	**Ionophores**											
IV	Lasalocid	10	4	1	15 (4%)	0%	4%	0%	Coccidiosis	6%	4%	0%
	Maduramicin	0	10	1	11 (3%)	0%	3%	0%	Coccidiosis	**9%**	**1%↓**	0%
	Monensin	28	45	39	112 (30%)	0%	30%	0%	Coccidiosis	**23%**	25%	**69%↑**
	Narasin	21	31	22	74 (20%)	0%	20%	0%	Coccidiosis	22%	18%	25%
	Narasin-nicarbazin	30	37	47	114 (31%)	0%	31%	0%	Coccidiosis	27%	34%	21%
	Salinomycin	35	50	56	141 (38%)	0%	29%	0%	Coccidiosis	**43%**	41%	**13%↓**
	**Chemical coccidiostats**											
N/A	Nicarbazin	34	40	47	121 (32%)	0%	32%	0%	Coccidiosis	**24%**	33%	**46%↑**
	Decoquinate	0	24	4	28 (8%)	0%	8%	0%	Coccidiosis	**16%**	**5%↓**	4%
	Clopidol	11	7	8	26 (7%)	0%	7%	0%	Coccidiosis	5%	8%	4%
	Diclazuril	7	0	2	9 (2%)	0%	2%	0%	Coccidiosis	5%	2%	0%
	Zoalene	3	3	3	9 (2%)	0%	2%	0%	Coccidiosis	3%	2%	2%
	Robenidine	0	1	3	4 (1%)	0%	1%	0%	Coccidiosis	1%	1%	0%
	*No medication in feed*	7	13	14	34 (9%)					8%	10%	6%

Roman numerals II to IV indicates categories if importance to human medicine as outlined by the Veterinary Drugs Directorate, Health Canada. N/A-not applicable; no VDD classification at the time of writing of this manuscript. GP–Growth promotion.

^¥^ For the temporal analysis, 2015 was compared to 2013 (initial surveillance year) and 2014 (previous year). Bold fonts represent significant temporal differences (*P*≤0.05) and the arrows (↓ or ↑) represent downward or upward directionality of the change.

^§^ For the seasonal variations, summer (grown between May and August) was compared to winter (grown between January to April) and fall (grown between September to December). Bold fonts represent significant seasonal differences (*P*≤0.05) and arrows (↓ or ↑) represent downward or upward directionality of the change.

**Table 3:** Period summary (2013–2015) of antimicrobial use via water in broiler chicken flocks, frequency, reasons for use and seasonal variations.

		2013	2014	2015^¥^	Total	Reasons for use		Diseases targeted	Seasonal variations^§^			Prescription^£^
		n = 97	n = 141	n = 135	n (%)	Treatment	Prevention		Summer	Fall	Winter	n
I	Enrofloxacin	2	0	0	2 (1%)	1%	0%	Neonatal diseases	2%	0%	0%	2
II	Apramycin sulfate	0	1	0	1 (<1%)	<1%	0%	Neonatal diseases	1%	0%	0%	1
	Amoxicillin	0	2	3	5 (1%)	1%	0%	Septicemia, airsacculitis	1%	1%	2%	5
	Penicillin G potassium	4	9	3	16 (4%)	3%	1%	Septicemia, necrotic enteritis	6%	4%	2%	10
	Penicillin-streptomycin	0	0	6	6 (2%)	0%	2%	Osteomyelitis	0%	3%	0%	2
III	Oxytetracycline-neomycin	0	0	1	1 (<1%)	<1%	0%	Septicemia, airsacculitis	0%	0%	0%	1
	Tetracycline	0	0	1	1 (<1%)	0%	<1%	Airsacculitis	0%	0%	0%	0
	Tetracycline-neomycin	0	4	0	4 (1%)	0%	1%	Septicemia, airsacculitis	1%	0%	6%	1
	Sulfamethazine	0	1	3	4 (1%)	1%	0%	Septicemia, coccidiosis	1%	1%	2%	3
	Sulfaquinoxaline	1	5	2	8 (2%)	2%	0%	Septicemia, coccidiosis	3%	1%	4%	2
	Sulfaquinoxaline-pyrimethamine	2	1	2	5 (1%)	1%	1%	Coccidiosis	1%	2%	0%	5
	No medication	90	121	114	325 (87%)				85%	88%	85%	

Roman numerals 1 to IV indicates categories if importance to human medicine as outlined by the Veterinary Drugs Directorate, Health Canada.

^¥^2015 was the referent year; for the temporal analysis (if drug was used in ≥10% of flocks), 2015 (current surveillance year) was compared to 2013 (initial surveillance year) and 2014 (previous year). Bold fonts represent significant temporal differences (*P*≤0.05).

^§^For seasonal variations, summer (referent season, pre-harvest sampling between May and August) was compared to winter (grown between January to April) and fall (grown between September to December).

^£^Veterinary prescription provided.

**Table 4:** Period summary (2013–2015) of antimicrobial use via *in ovo* or subcutaneous route in broiler chicken flocks, frequency, reasons for use and seasonal variations.

	Antimicrobial	2013	2014	2015^¥^	Total	Diseases targeted	Seasonal variations		
		n = 99	n = 143	n = 136	n (%)		Summer^§^	Fall	Winter
I	Ceftiofur	31	9	0	40 (11%)	APEC	8%	11%	14%
II	Gentamicin	3	7	13	23 (6%)	APEC, *Salmonella* spp.	3%	7%	6%
	Lincomycin-spectinomycin	24	34	40	98 (26%)	APEC, enteric diseases	**19%**	**31%↑**	16%
	No medication used at the hatchery	**42**	92	**82↑**	216 (57%)		**70%**	**51%↓**	64%

Roman numerals I to II indicates categories if importance to human medicine as outlined by the Veterinary Drugs Directorate, Health Canada. APEC-avian pathogenic *Escherichia coli*

^¥^ Referent; for the temporal analysis, 2015 was compared to 2013 (initial surveillance year) and 2014 (previous year). Values in bold fonts represent significant temporal differences (*P*≤0.05). arrows (↓ or ↑) represent downward or upward directionality of the change.

^§^ Summer-referent; for the seasonal variations, summer (May and August) was compared to winter (January to April) and fall (September to December). Values in bold fonts represent significant seasonal differences (*P*≤0.05) and arrows (↓ or ↑) represent downward or upward directionality of the change.

#### Route of administration

Over the three years of surveillance, 91% of flocks were exposed to antimicrobials via the feed while the remaining 9% were not exposed to any antimicrobials, including ionophores and chemical coccidiostats. Only 13% of producers reported the use of antimicrobials via water.

#### Feed—Specifics regarding antimicrobial classes

A total of 9 different antimicrobials (belonging to 8 classes) and 12 different coccidiostats were reported. Among the antimicrobials added in feed, bacitracin was the most frequently used with 53% (198 medicated flocks/373 flocks with feed-level information) of flocks using bacitracin. Virginiamycin was the second most frequently reported antimicrobial used in 2013 (Table 2). However, the number of flocks with reported virginiamycin use significantly decreased (*P≤0*.*0001*) between 2014 (20%, 28/141) and 2015 (16%, 22/135) (Table 2). This decline in virginiamycin use corresponded to a significant increase (*P≤0*.*0001*) in the use of avilamycin (2014: 23%, 33/141) (2015: 34%, 46/135). For all other antimicrobials and coccidiostats the reported frequency of flock use and overall relative ranking remained stable over the three years.

Thirty-eight percent (141/373) of flocks reported the use of salinomycin, which was the most frequently used ionophore (Table 2). Narasin-nicarbazin was the second most commonly reported ionophore combination followed by monensin (Table 2). Among the chemical coccidiostats, nicarbazin was reported most frequently and was used by 32% (121/373) of flocks (Table 2). The next most common chemical coccidiostat was decoquinate with only 8% (28/373) of flocks reporting the use of this drug (Table 2).

#### Reasons for use (feed)

Most broiler rations typically contained a combination of two or more products, one to target *Clostridium perfringens*, the causative agent of necrotic enteritis, and one or more products against *Eimeria* spp., the causative agent of coccidiosis (Table 2). Trimethoprim-sulfadiazine, was an exception and was used for the treatment of systemic (e.g., colibacillosis) and localized diseases (e.g., airsacculitis and osteomyelitis) (Table 2).

#### Seasonal variations (feed)

Avilamycin and penicillin G procaine were used more frequently in the fall compared to the summer months (referent season, *P* = 003 for both antimicrobials), while tylosin was used more in the winter compared to the summer months (*P* = 0.0136) (Table 2). There were also seasonal variations observed with the use of coccidiostats. In the summer, the more frequently reported coccidiostats were salinomycin (*P* = 0.0006, compared to winter), maduramicin (*P* = 0.0025 compared to fall) and decoquinate (*P* = 0.0017 compared to fall), while in the winter, the more frequently reported coccidiostats were monensin (*P*<0.0001 compared to summer) and nicarbazin (*P* = 0121 compared to summer) (Table 2).

#### Water—Specifics regarding antimicrobial classes

There were 11 antimicrobial ingredient/combination products reported (Table 3). Penicillin was the most frequently used product with 4% (16/373) of flocks reporting use, followed by penicillin-streptomycin and sulfaquinoxaline, each with 2% (8/373) of flocks reporting use (Table 3) There were a limited number of producers reporting the use of enrofloxacin (2013, n = 2); fluoroquinolones are not labelled for use in poultry in Canada.

#### Reasons for use

Unlike antimicrobials administered via feed, which are largely utilized for prevention of enteric diseases, antimicrobials administered via the water were administered to treat systemic and localized diseases (Table 3). In most cases, a veterinary prescription was provided to the producer (60% of all treatments), particularly when the antimicrobial use was deemed to be extra label (Table 3).

#### Seasonal variations (water)

There was no seasonality to the use patterns identified (Table 3).

#### Injections—Specifics regarding antimicrobial classes

At the hatcheries, there were three antimicrobials/antimicrobial combinations documented: ceftiofur, gentamicin, and lincomycin-spectinomycin (Table 4). Over the three surveillance years, 43% of the flocks surveyed were medicated at the hatchery level. There were reports of ceftiofur use prior to 2015, however the number of producers reporting ceftiofur use significantly decreased from 31% (31/99) in 2013 to 6% in 2014 (9/143, *P*≤0.05) (Table 4). The number of flocks that were not medicated increased in 2015 (82/136, *P* = 0.0071) compared to 2013 (44/99).

#### Reasons for use (injectables)

The reported use of gentamicin, approved for subcutaneous use in chickens to treat avian pathogenic *Escherichia coli* (APEC) and *Salmonella* spp., and lincomycin-spectinomycin, a product that is currently an ELDU in chickens, has increased in use to treat these neonatal infections over the last two years (Table 4).

#### Seasonal variations (injectables)

The use of lincomycin-spectinomycin was significantly higher during the fall months (31%) compared to the summer months (19%, *P*≤0.001) (Table 4). When all surveillance years were analyzed together there were more flocks that were not medicated at the hatchery during the summer months compared to the fall months (*P* = 0033) and winter months (not significant).

#### Quantity of antimicrobials used

**Fig 2:**
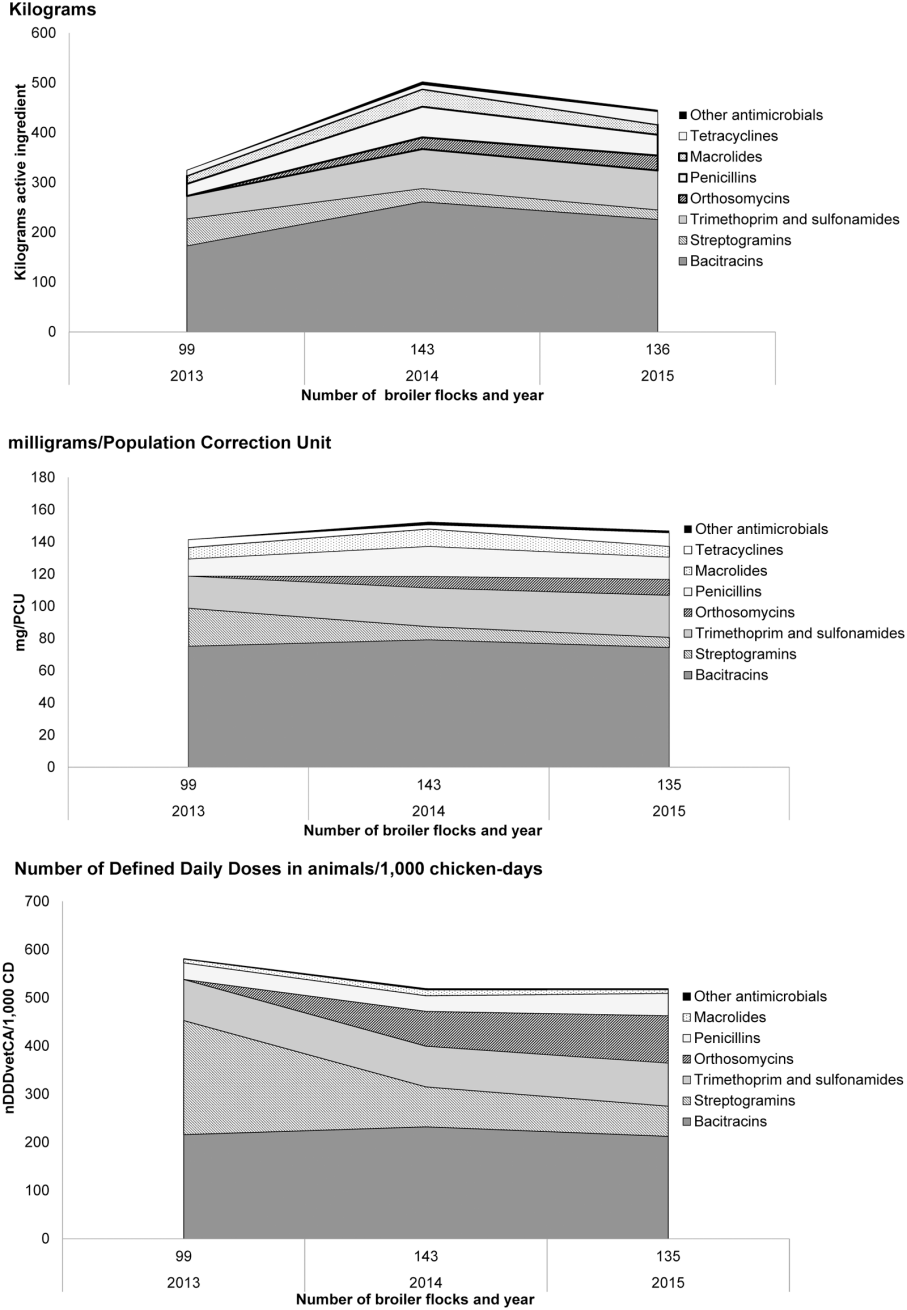
Comparison of trends in antimicrobial use in broiler chicken flocks using three quantitative metrics (n = 378 flocks), 2013–2015.

Including ionophores, chemical coccidiostats and antimicrobials labelled for growth promotion, antimicrobials administered via feed represented the greatest route of administration/exposure to broiler chicken flocks (95% of kg used, 3,000/3,144 kg). The quantities of products administered via water were less (5%, 142/3,144 kg), and the remaining <1% were those products that were administered via *in ovo* or subcutaneous injections at the hatchery level (Table 1). Fig 2 summarizes the quantities of antimicrobials used from 2013–2015 (n = 378 flocks; 8.6 million birds).

#### Kilograms

There was a marked increase in total kg of active ingredient reported between 2013 (325 kg) and 2014 (503 kg, 55% increase from 2013), which then modestly declined to 446 kg in 2015 (11% decrease) (Fig 2 and S3 Table). As shown in Fig 2, the percentage of some antimicrobial classes varied from year to year due in part to changing use patterns, notably, the decline in total kg of virginiamycin that corresponded to a rise in kg of avilamycin. The top three antimicrobial classes measured by kg for the period (2013–2015) included bacitracins (660 kg), trimethoprim and sulfonamides (205 kg) and penicillins (128 kg).

#### Milligrams/PCU

Unlike the trends depicted by kg of active ingredient between 2013 and 2014, the mg/PCU only changed marginally. There was an increase in overall mg/PCU by 8% between 2013 and 2014 (142 mg/PCU to 152 mg/PCU) followed by a decrease of 4% in 2015 (147 mg/PCU) (Fig 2). The magnitude of change by antimicrobial class varied more considerably. The streptogramins decreased by 66% between 2013 and 2014; and penicillin increased by 76% between 2013 and 2014 (Fig 2). Similar to total kg of antimicrobials used, the top three antimicrobial classes measured by mg/PCU were bacitracins, trimethoprim and sulfonamides and penicillins (Fig 2 and S3 Table).

#### Number of DDDvetCA/1,000 CD

The nDDDvetCA/1,000 CD decreased by 11% between 2013 and 2014 and increased by 2% between 2014 and 2015 (Fig 2). Overall, the magnitude of change among antimicrobial classes varied. The nDDDvetCA/1,000 CD for streptogramins showed the greatest magnitude of change between 2013 and 2014 (Fig 2). In 2013, even though bacitracins were ranked as the largest use in terms of kg of active ingredient and as mg/PCU, the nDDDvetCA/1,000 CD for streptogramins (assigned DDDvetCA_mg_ = 2.9) surpassed that of the bacitracins (assigned DDDvetCA_mg_ = 10.1) (S2 Table). The top three classes with the largest nDDDvetCA/1,000 CD for the period were bacitracins, streptogramins, and trimethoprim and sulfonamides (Fig 2 and S3 Table).

Overall, the temporal variations show differences depending on the AMU metric. The kg of active ingredient metric indicated an increase in use since 2013, the mg/PCU indicated overall use remained relatively stable, and nDDDvetCA/1,000 CD indicated that overall use decreased since 2013 (Fig 2).

#### Average treatment days/cycle

**Fig 3:**
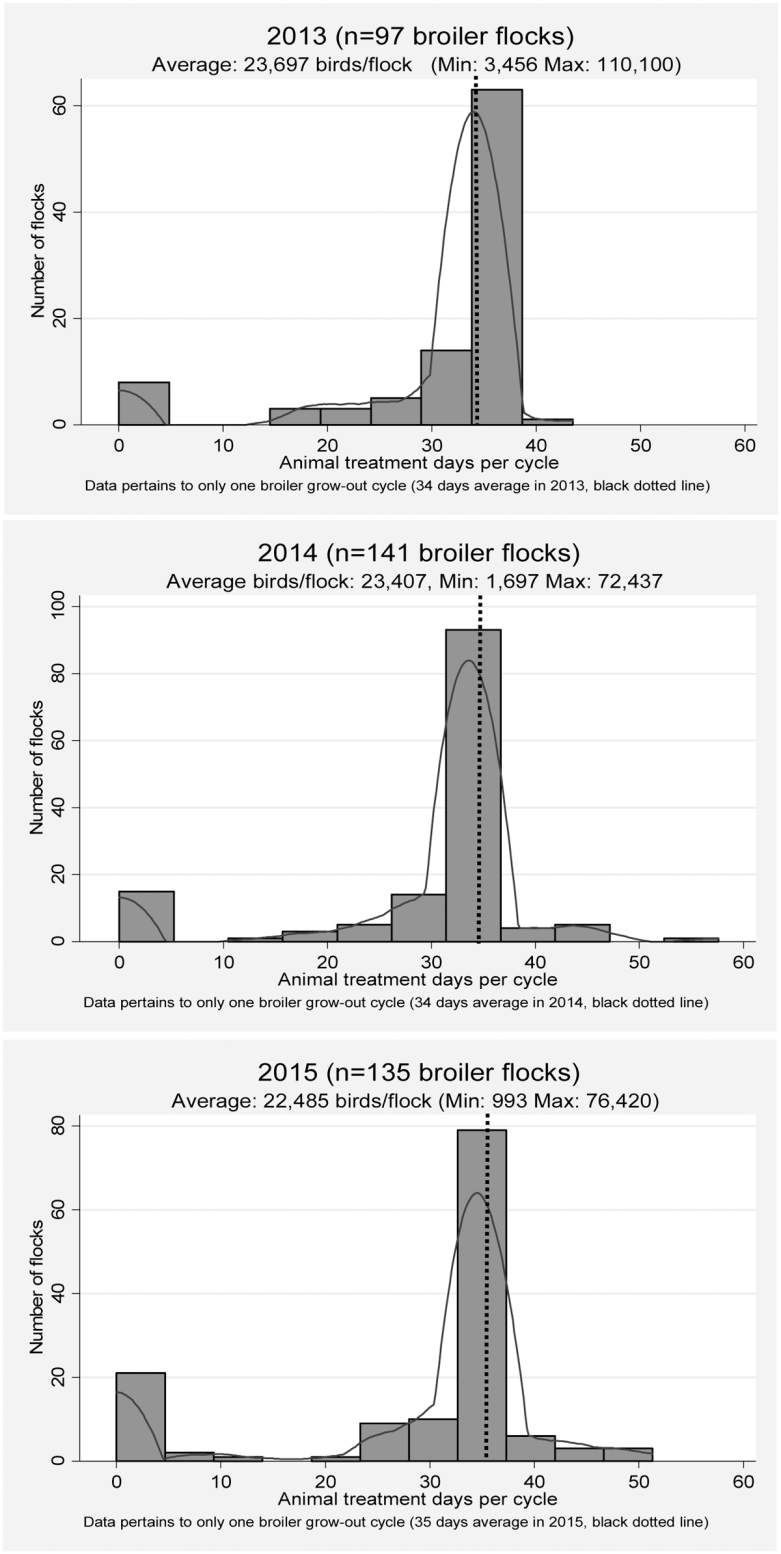
Frequency distribution of animal treatment days (ATC)/cycle per broiler flock for one grow-out cycle (n = 373 flocks, 2013–2015).

The frequency distribution of ATD/cycle is shown in Fig 3. The median ATD/cycle during the study timeframe was 34 ATD/cycle. Flocks with zero ATD/cycle corresponded to the flocks raised without any antimicrobials including coccidiostats and organic flocks. The flocks with the maximum ATD/cycle were the flocks raised as heavy broilers/roasters (59 ATD/cycle). The vast majority of the flocks were fed with medicated rations within the 50-percentile (34 ATD/cycle) and 70- percentile (35 ATD/cycle). These ATD/cycle values were equal to the age of the flocks at time of the pre-harvest visit (black dotted lines). The distribution of flocks was relatively consistent from year to year and there was no marked shift to either the left or right side of the distribution curve observed over time (Fig 3).

### Relative proportion of antimicrobials used in broilers, swine and food animals

**Fig 4:**
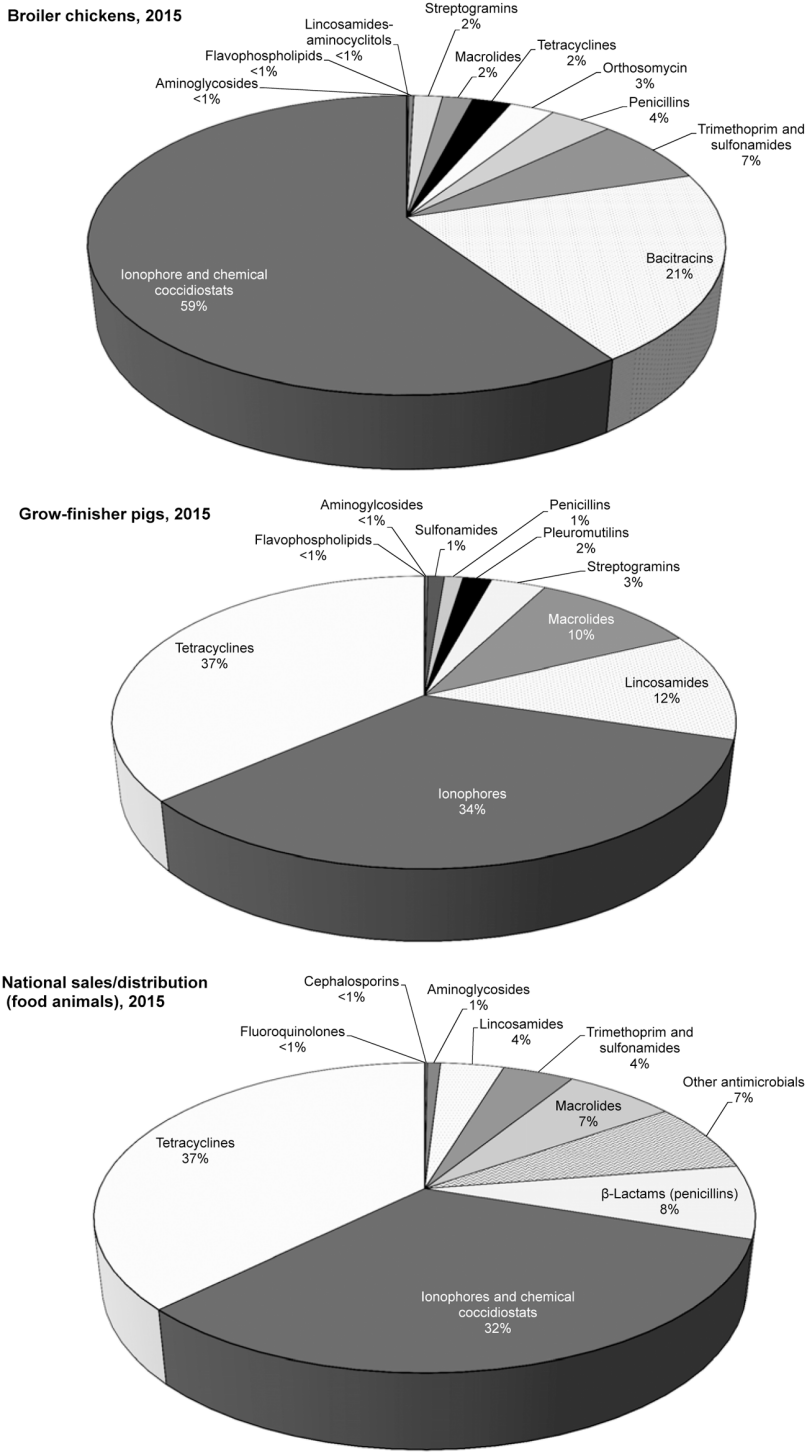
Percentages of the quantities of antimicrobials (kg) in broiler chickens, grow-finisher pigs and the national distribution data in food animal species in 2015. *Other antimicrobials in broilers included classes used in limited quantity (<2 kg) including third generation cephalosporins, fluoroquinolones, aminoglycosides and aminocyclitol-lincosamides. In the national sales/distribution data, antimicrobial classes are based on accounting rules to protect confidential business information of the drug manufacturers. The ‘other antimicrobials’ for the national sales/distribution data included avilamycin, bacitracins, bambermycin, chloramphenicol, chlorhexidine gluconate, florfenicol, fusidic acid, nitrofurantoin, nitrofurazone, novobiocin, polymixin, tiamulin, and virginiamycin. Grow-finisher herds included in the surveillance were close to market pigs and weighed ≥ 80 lbs (145 kgs).

The percentage of antimicrobial classes in kg consumed by the broiler flocks was compared to the grower-finisher swine herds included in CIPARS farm surveillance, and the national distribution data for food animals from CAHI for 2015 (Fig 4). Unlike in a similar figure (Fig 2), data presented in Fig 4 includes ionophores and chemical coccidiostats. The largest relative AMU (59%) in kg active ingredient was ionophores and chemical coccidiostats followed by bacitracins (21%), and trimethoprim and sulfonamides (7%) (Fig 4, S4 Table). In grower-finisher swine herds (feed only data was available), there were similarities in the classes of antimicrobials used but the relative proportion of some classes markedly differed, such as for tetracyclines (Fig 4, S4 Table). Additionally some reported antimicrobials used via feed in swine were not used at all in broiler chickens, such as lincosamides and pleuromutilins. The relative ranking of antimicrobials at the national distribution level for food animals was also different from broiler chickens; tetracyclines had the highest quantity (37%), followed by ionophores and chemical coccidiostats (32%) and beta-lactams (penicillins, 8%) (Fig 4).

The temporal trends in mg/PCU (excluding coccidiostats) between animal species are presented in Table 5. Overall mg/PCU estimates are relatively higher in grow-finisher swine herds and the national food animal population then those of broiler chickens. It is important to note that these estimates of mg/PCU represent use for one grow-out cycle for grower-finisher pigs and broiler chickens, whereas the estimate of mg/PCU for the national distribution data is based on the annual reported quantity of antimicrobials distributed for sale for food animal uses.

**Table 5:** Temporal variations in milligrams per Population Correction Unit (mg/PCU) of antimicrobials used in broiler chickens, grower-finisher pigs and the national distribution data for all food animal species in 2014–2015.

	Broiler chickens	Grow-finisher pigs	National sales/distribution
Year	(mg/PCU)	(mg/PCU)	(food animals), mg/PCU
2013	142	146	171
2014	152	165	171
2015	147	176	183

Grow-finisher pigs included in the surveillance were close to market pigs and weighed ≥ 80 lbs (145 kgs).

The national sales/distribution data represent quantities of antimicrobials reported by manufacturers and distributors in Canada. These latter data do not include antimicrobials imported under the 'own use' provision or imported as active pharmaceutical ingredients used in compounding.

## Discussion

Data from CIPARS have addressed a previous Canadian knowledge gap by providing an overview of AMU reported by sentinel broiler farms from five provinces between 2013 and 2015. There were at least 14 different classes of antimicrobials given via injections, feed or water. Analysis of various quantitative metrics showed temporal variations in overall AMU and by individual class of antimicrobials, demonstrating that the metric reported can impact data interpretation.

One of the goals of AMU surveillance programs is to measure the impact of AMU interventions [4]. As CIPARS is an integrated surveillance program capturing both AMU and AMR information from the same sentinel farms, it can provide information to assess the impact of risk reduction strategies, such as the poultry industry banning of the preventive use of ceftiofur. Declining trends in the use of ceftiofur, as reported in this document, complimented with declining ceftiofur resistance trends among *E*. *coli* and *Salmonella*, are consistent with the timing of the May 2014 industry led intervention eliminating the preventive use of antimicrobials critically-important to human medicine, such as third generation cephalosporins and fluoroquinolones [20,21]. Ceftiofur has been used historically at the hatchery to prevent neonatal diseases caused by avian pathogenic *E*. *coli* (APEC), the classical causative agent of various diseases in poultry species such as yolk sac infections and septicemia [22,23]. Diseases associated with APEC are routinely diagnosed in Canadian flocks [24] and are a concern because of increased mortality due to serious sequelae of some of these infections [23].

CIPARS observed another important impact of the intervention eliminating the use of ceftiofur. Following the industry intervention, there was an increase in the use of gentamicin and lincomycin-spectinomycin, with simultaneous increase in resistance to gentamicin (data not shown, [25]). These drugs may have been used to replace ceftiofur to address ongoing disease pressures or production-related issues (i.e., breeder flock health). These data are therefore a good example of how on-going farm surveillance can be used to monitor the potential unexpected consequences of an intervention.

CIPARS documented AMU practices intended to control specific diseases of broiler chickens. Most of the antimicrobials used in feed were for the prevention of enteric diseases, such as coccidiosis and necrotic enteritis. Based on the estimated quantity of medicated feeds, various antimicrobial classes that are efficacious against *C*. *perfingens* (e.g., bacitracins, streptogramins, penicillins, macrolides, orthosomycins) were the key drivers of AMU in the broiler flocks surveyed. Certain antimicrobial classes including bacitracins and streptogramins are no longer permitted for growth promotion use in Europe [26] which might explain the comparatively lower overall usage in certain European countries or a smaller relative proportion of the use of certain classes (e.g., bacitracins) [5].

The CIPARS data demonstrated a shift in treatment options for necrotic enteritis, where the use of virginiamycin was replaced by avilamycin in some instances. This shift in AMU for necrotic enteritis may have been due to the availability of a new product (avilamycin), to preserve the efficacy of an existing product, its compatibility with coccidiostats, and other factors (i.e., producer, veterinarian and feedmill preference based on previous flock performance using this product). However, there is no national surveillance of AMR in *C*. *perfringens* currently, making the reason for the switch in AMU difficult to assess. Susceptibility of clinical *C*. *perfringens* isolates in one province (Ontario) has indicated moderate to high level of resistance to commonly used drugs (e.g., bacitracin, tetracycline) [27,28], but more data and more recent AMR profiles need to be acquired to measure the clinical implications of changing AMU patterns for necrotic enteritis control.

For coccidiosis prevention in Canadian chicken flocks, salinomycin was the most frequently reported coccidiostat used. Overall, in Canada, coccidiostats contributed to 53% of all antimicrobials used in broiler chicken flocks. However, we observed seasonal variations in some of the most frequently used drugs that may reflect rotational programs to manage coccidiosis. Notably, the use of nicarbazin was more frequent in the colder seasons, which was expected due to the negative health impact of this drug in the event of high temperature and humidity during the warmer seasons [29]. Other factors not captured via the CIPARS broiler chicken questionnaire, such as costs or preferences of the feedmill, veterinarian, or producer, may have influenced frequency of certain coccidiostat use.

Ionophores and chemical coccidiostats are deemed not medically important [30,31] and are often not reported in national AMU surveillance programs. Peer-reviewed literature has noted the public health implications of ionophore use, such as the potential linkage between transferrable decreased susceptibility to narasin and maintenance of vancomycin resistance in Enterococci [32]. The risks of ionophore (narasin, monensin and salinomycin) resistance development among Enterococci/gut flora have been cited [33,34] warranting further research to assess their impact on the environment (e.g., residues) and in humans. CIPARS continues to monitor the frequency of use and quantity of coccidiostats, because of their clinical relevance, such as anti-clostridial activity of some ionophores in subclinical necrotic enteritis [35]. Similarly, Denmark, for the first time since 2004, started to report the consumption of ionophores and chemical coccidiostats [33]. In general, coccidiostats used in Canadian flocks were similar to those used in broiler chickens in Denmark [33]. Non-antimicrobial interventions and targeted reduction approaches to control both coccidiosis and necrotic enteritis will contribute to the reduction of the total kg of AMU in broiler chickens in Canada.

For administration via water, the farm questionnaire monitored prescription behaviours/veterinary consultation, an important aspect of a valid veterinary-client-patient-relationship. Our data indicate that 60% of the producers that treated their flock via water obtained a veterinary prescription. This level is expected to improve when upcoming legislations on enhanced veterinary oversight and prescription only use will take effect. The feed section of the questionnaire currently does not collect information regarding prescriptions/veterinary consultations for medicated feed, but collecting this information would add value to the surveillance program (i.e., monitoring the impact of upcoming AMU policy changes) [15,16].

There were no observed seasonal differences with regard to water treatment, but for hatcheries it appeared that medications were more frequently administered to chicks hatched during the colder months (fall and winter); somewhat expected due to the barn/environmental conditions and operational factors (e.g., stressors to eggs or chicks) during these seasons.

Similar to the estimation approach used in a study of swine herds [36] with few modifications, the CIPARS farm program (swine, broilers, turkeys) used feed and water standards and simple regression to estimate the quantity of feed and water consumed. This approach reduced the potential for overestimating (e.g., feed delivered vs. feed consumed) or underestimating (e.g., missing feed delivery receipts, recall bias) AMU; however, barn-level factors influencing feed and water quantity, including ambient temperature, humidity, feed and water quality, and mechanical failures in the feed and water lines influencing level of consumption in birds were unaccounted for in quantitative estimates provided by this method.

The various AMU metrics will allow CIPARS to compare usage with other countries. In Europe, the mg/PCU metric identifies high and low users [5] and was one of the indicators used to monitor legislative changes in the Netherlands [37]. Adjusting the quantity of antimicrobials for population and weight provides essential context to interpret the quantities of antimicrobials used. Somewhat obviously, there was a remarkable increase in kilograms of active ingredient used when the sample size increased between 2013 and 2014, but when the quantity of antimicrobials was adjusted for population and weight, the resultant change in mg/PCU was relatively small. For adjusting the average weight at treatment, we used the ESVAC standard weight of 1 kg/bird, which was similar to the average weight of birds at treatment in our Canadian dataset (0.9 kg/bird).

The nDDDvetCA/1,000 CD metric accounted for the average daily dose (potency) of products available in Canada. Our approach in assigning DDDvetCA_mg_ involved stratification by route of administration (e.g., feed and water), a modification of the DDDvet approach described by ESVAC (e.g., one DDDvet assigned for any oral pharmaceutical form in ESVAC). This greater stratification permitted detection of shifts in use from one route of administration to another, such as potential shift from feed uses that involve lower dosage but prolonged duration of exposure to prevent disease, to water uses that involved therapy of specific diseases diagnosed at higher average daily doses and shorter duration of exposure. The nDDDvetCA/1,000 CD accounted for population and days in the observation period (i.e., equivalent to average growing period), also can permit detection of differences in AMU over time, between antimicrobial classes and possibly animal species (in the future). This metric compliments and provides a more accurate depiction of true bacterial selection pressure than mg/PCU measurements for monitoring AMU over time and the impact of AMU reduction initiatives in a Canadian context. The European Medicines Agency’s draft ‘Guidance on Provision of Data on Antimicrobial Use by Animal Species from National Data Collection Systems’[6] described the use of number of DDDvet/PCU for reporting and communicating AMU by European member countries to the European Medicines Agency. Reporting by number of DDDvetCA/PCU in addition to mg/PCU facilitates armonized reporting of AMU with other countries and comparison of the level of use over time.

Another metric used was ATD/cycle, modified slightly from the method described elsewhere ([10]. Although this metric does not account for biomass or potency of the antimicrobial, this information is more accessible to producers and veterinarians and allows for direct comparison of the length of treatment days (AMU exposure) during the grow-out cycle between farms and thus, could be applied for benchmarking purposes. At the industry level, this metric could be used for monitoring the impact of AMU reduction initiatives and complement the other quantitative metrics described above.

Differences in AMU/antimicrobial distribution in broiler chickens, swine, and the general food animal population (kg, mg/PCU) are reflective of disease pressures, drug product availability for different animal species, and other production/operational factors specific to an animal population. Over time, monitoring of the quantity of AMU across multiple species will measure broad overall AMU reduction strategies implemented at a regional or national level. The CAHI national distribution data showed more diverse antimicrobial classes not reported used, or used in very small quantities in poultry (e.g., beta-lactams, fluoroquinolones, tetracyclines). When compared to 2014 data from Europe [5], the 2015 Canadian mg/PCU in production animals are relatively higher than average; Canada was 25 out of 30 countries, when ranked from smallest to highest mg/PCU.

CIPARS on-farm AMU surveillance provided previously unavailable information about AMU in broiler chickens, detected temporal AMU changes, and measured the impact of industry-led interventions towards AMU reduction. Contextualizing local disease pressures (e.g., data obtained through the farm surveillance questionnaire and other industry sources such as veterinary reports) and changes in AMR trends/patterns will compliment AMU measurements to inform targeted interventions to reduce AMR risks.

## Supporting information

S1 Table Broiler chicken surveillance framework, Canadian Integrated Program for Antimicrobial Resistance Surveillance. (DOCX)

S2 Table Assigned Defined Daily Doses in broiler chickens in Canada (DDDvetCa). A manuscript detailing the DDDvetCA assignment for broiler chickens is in preparation. **ESVAC**-European Surveillance of Veterinary Antimicrobial Consumption (European Medicines Agency, European Union). **ELDU**—Extra-label Drug Use; this antimicrobial is not approved for use in broiler chickens in Canada. **DDDvetCA**—assigned Defined Daily Doses for animals (chickens), Canadian values (DDDvetCA in mg/kg body weight = DDDvetCA mg/broiler chicken; the ESVAC standard weight of 1 kg broiler was used). **DDDvetEu**—assigned Defined Daily Doses for animals, European values (Source: http://www.ema.europa.eu/docs/en_GB/document_library/Scientific_guideline/2015/06/WC500188890.pdf). **TMP**—Trimethoprim-sulfadiazine combination drug. **n/a**-not applicable or unavailable; no ESVAC DDDvet values that are currently assigned for these antimicrobials/coccidiostats and products (some active ingredient are no longer permitted for use in broiler chickens). Active ingredients with underscore are combination products; for that row, the DDDvetCA is for the bolded drug when it is in combination with the unbolded drug (e.g. for Lincomycin_Spectinomycin–the DDDvetCa is for Lincomycin when in combination with spectinomycin; and for Spectinomycin_lincomycin–the DDDvetCa is for spectinomycin when in combination with lincomycin.) Unless indicated [Growth promotion, (GP only)], the average dose for each antimicrobial was based on prevention and treatment label claims. The average dose is the sum of all unique doses for prevention and treatment divided by the total number of unique doses. The “Oral” route of administration for ESVAC includes feed, water and oral bolus doses; parenteral in the ESVAC values is equivalent to Canada’s injectable.
(XLSX)

S3 Table Values used in Fig 2: Kilogram, milligrams per Population Correction Unit (mg/PCU) and number of Defined Daily Doses in animals/1,000 chicken-days at risk (nDDDvetCA/1,000 CD). (XLSX)

S4 Table Values used in Fig 4, Pie chart, kilograms antimicrobials in broiler chickens and grower-finisher pigs, 2015. The national sales and distribution data for production animal is provided by the Canadian Animal Health Institute (CAHI). Raw data may not be available as CAHI provides the information according to a "3 company accounting rule" established by CAHI to comply with the European Union and the United States’ anti-competition regulations. CAHI added in some cases a "90% rule" to be sure not to infringe the regulations in the United States. For more information regarding the national sales and distribution data, please contact Dr. Carolee Carson (carolee.carson@phac-aspc.gc.ca).
(XLSX)

S1 Fig Water estimation using simple logistic regression and integral calculus. Water consumption estimates were obtained from the Nutreco Canada Inc.-Shurgain standards daily water consumption chart and a plot of intake in liters/bird/day was created (i.e., similar to the feed estimation methods). Regression parameters were calculated within Microsoft Excel by using the plotted water intake curve. As in feed, a minimum R-square value of more than 0.99 was required to be considered a good fit therefore to obtain the best fitting regression values the water consumption curve was divided into 3 segments.
(TIFF)

S1 Text Definitions and Canadian industry information used in the manuscript. (DOCX)
