# Supplementary material for: Antimicrobial use surveillance in broiler chicken flocks in Canada, 2013-2015
Source: PLoS One. 2017 Jun 28;12(6):e0179384. doi: 10.1371/journal.pone.0179384 (PMC5489168; doi:10.1371/journal.pone.0179384)
Supplement: S1 Table — (DOCX) [file pone.0179384.s001.docx]

**S1 Table. Broiler chicken surveillance framework, Canadian Integrated Program for Antimicrobial Resistance Surveillance (CIPARS)**

| **Variable** | **Description** |
| --- | --- |
| **Surveillance objectives** | 1. Primary Objectives  - Provide representative qualitative and quantitative hatchery and farm data on antimicrobial use and resistance at the national and regional level.  1. Secondary Objectives  - Investigate associated trends in antimicrobial use (AMU) and resistance (AMR) at a national and regional level.  1. Long-term objectives  - Provide sound data for human health risk assessments.   Provide data to industry to help support science-based decisions to reduce AMR. |
| **Legal basis for collection of use in broilers** | Voluntary |
| **Administration** | Canadian Integrated Program for Antimicrobial Resistance Surveillance |
| **Data collection structure** | Sample survey |
| **Data collector** | Poultry veterinarians   - **Justification:** Veterinary-patient-client-relationships (VCPR) for AMU; technical/clinical background of the veterinarian may improve data quality; client trust in the veterinarian; knowledge on biosecurity and local/regional disease conditions. |
| **Sampling frame/sentinel farm allocation** | Approximately 145 sentinel broiler flocks from 145 unique sentinel broiler farms per year in the 5 poultry-producing provinces: British Columbia, Alberta, Saskatchewan, Ontario, Québec   - Allocation per province is proportional to the total broiler population slaughtered per year |
| **Sampling strategy** | One randomly selected flock from a single unit within a farm.   - A flock(epidemiological unit of interest) is defined as a group of birds hatched and delivered to a single unit (barn, floor or pen) at approximately the same day within a farm. A farm is defined as a broiler facility that has barns dedicated to broiler production under the supply management (Source: <http://www.chickenfarmers.ca/how-we-do-it/what-is-supply-management/>). The farm site can have multiple barns and multiple floor levels or pens within a barn. Management practices and biosecurity levels may differ from farm-to-farm. - **Justification:** Logistics and practicality of collecting barn-level information (homogeneity of AMU within the farm since the same feedmill and hatchery supply feed and chicks, respectively, to the producer; antimicrobial resistance profiles are expected to be similar within the farm). The unit of interest is consistent with published studies on broiler AMR and pathogen recovery at pre-harvest stages.   Frequency of farm visit is one broiler grow-out cycle per flock per farm per year.   - **Justification:** Sustainability of the farm program (budget) and ability to link AMR and farm-level AMU (overall surveillance program considerations). |
| **Sentinel farm enrolment and selection criteria** | 1. **Random or purposive farm selection**:    1. Selection within province to represent the geographical distribution of broiler producers;    2. Where possible, sentinel farms should represent a diversity of chick sources to ensure that not one broiler chick supplier (hatchery) is over-represented    3. Should also represent a diversity of feed suppliers to ensure that not one feedmill is over-represented;    4. Enrolled by veterinarians (serve as confidentiality screen);    5. Veterinarians will satisfy inclusion/exclusion criteria set below;    6. Sentinel farms selected should also be reflective of their veterinary practice (e.g., varies by level of flock management: poor to excellent performing flocks, volume of chicks placed: low to high flock population, various level of biosecurity compliance). 2. **Inclusion criteria:**    1. Level of participation by the producer;    2. Safe Safer Safest/On-farm Food Safety Program compliant;    3. Quota-holding producer (≥14,000 units of quota, one unit corresponds to ~13 kgs. Source: <https://www.ontariochicken.ca/Farmer-Member-Resources/Quota-Info.aspx> );    4. Antimicrobial free broilers and organic farms (selected based on the veterinary practice profile);    5. Floors that have chicks that are sourced from imported breeder flocks (US). 3. **Exclusion criteria:**    1. Niche market production (Black Silkies);    2. Backyard flock/small flocks (non-quota farms/artisanal chickens. Source: <https://www.ontariochicken.ca/Programs/Artisanal-Chicken-Program.aspx>). |
| **Implementation** | 1. Veterinarians/field workers distribute their flock sampling over the different seasons or three sampling periods (winter, summer and fall). Each season corresponds to at least two quota periods in the Chicken Farmers of Canada’s 8-week quota allocation calendar (Source: <http://www.chickenfarmers.ca/wp-content/uploads/2015/07/Allocation-Calendar.pdf>). This sampling scheme is intended to distribute sampling over the year to describe any potential seasonal variations that could influence AMU decisions. 2. Veterinarians, at their own convenience will schedule their flock visits but ensures that sampling for flocks allocated is distributed across the year.    - This ensures that AMU and AMR data heterogeneity (i.e., not clustered in one season or quota period). |
| **Variables collected** | Farm and flock-level epidemiological information (no personal farm identifiers provided):   - Information pertaining to the chicks delivered (hatchery code, domestic or imported source); - Flock characteristics: strain, type of production (e.g., conventional, antibiotic free, organic ) - Total birds delivered, total birds at the time of the farm visit, farm capacity, quota period. - **Justification:** data to assess representativeness of flocks and farm selection by the veterinarian.   Antimicrobials administered via *in ovo* or subcutaneous at the hatchery:   - Final dose per hatching egg or chick; - Proportion of hatching eggs or chick medicated; - Reasons for use.   Antimicrobials administered via feed:   - Feed delivery receipts and accompanying documents (usually the feed formulation with the level of drug is available accompanied by the prescribing veterinarian consulted by the feedmill); - Level of drug in the feed (antimicrobials and coccidiostats); - Type of ration (starter, grower, finisher); medicated or non-medicated; - Feeding duration for each ration (age started and age ended for each ration); - Reasons for use; - **Justification:** data to estimate total kg used in the absence of information pertaining to the number of packages per veterinary medicinal product (VMP) or type and total kilograms of the feed premix used by the feed mill for the flock/farm (reporting mechanism is currently unavailable).   Antimicrobials administered via water:   - Product name; - Inclusion rate in grams per liter of drinking water; - Duration of water treatment (age started and age ended for each treatment); - **Justification:** data to estimate total kg used via water in the absence of the number of packages per VMP used on the unit of interest.   Other information:   - Vaccines administered at the hatchery and on farm; - Basic biosecurity information relevant to pathogen prevalence; - Animal health information (e.g., diseases diagnosed); - Zootechnical additives. - **Justification**: Data to complement AMU; used in risk factor studies/research to contribute to the understanding of AMU-AMR. |
| **Reporting** | Only aggregated AMU information is included in the CIPARS annual report/publications/communication products. A knowledge translation and exchange meeting with the poultry industry is scheduled once a year or as needed. |

The CIPARS broiler chicken surveillance framework (including the AMR component) was developed in 2009 to 2011 in consultation with an expert committee/working group that consisted of poultry veterinarians, provincial and national chicken marketing board representatives and other experts (e.g., academia).
