# Supplementary figures and images for: Antimicrobial use surveillance in broiler chicken flocks in Canada, 2013-2015
Source: PLoS One. 2017 Jun 28;12(6):e0179384. doi: 10.1371/journal.pone.0179384 (PMC5489168; doi:10.1371/journal.pone.0179384)

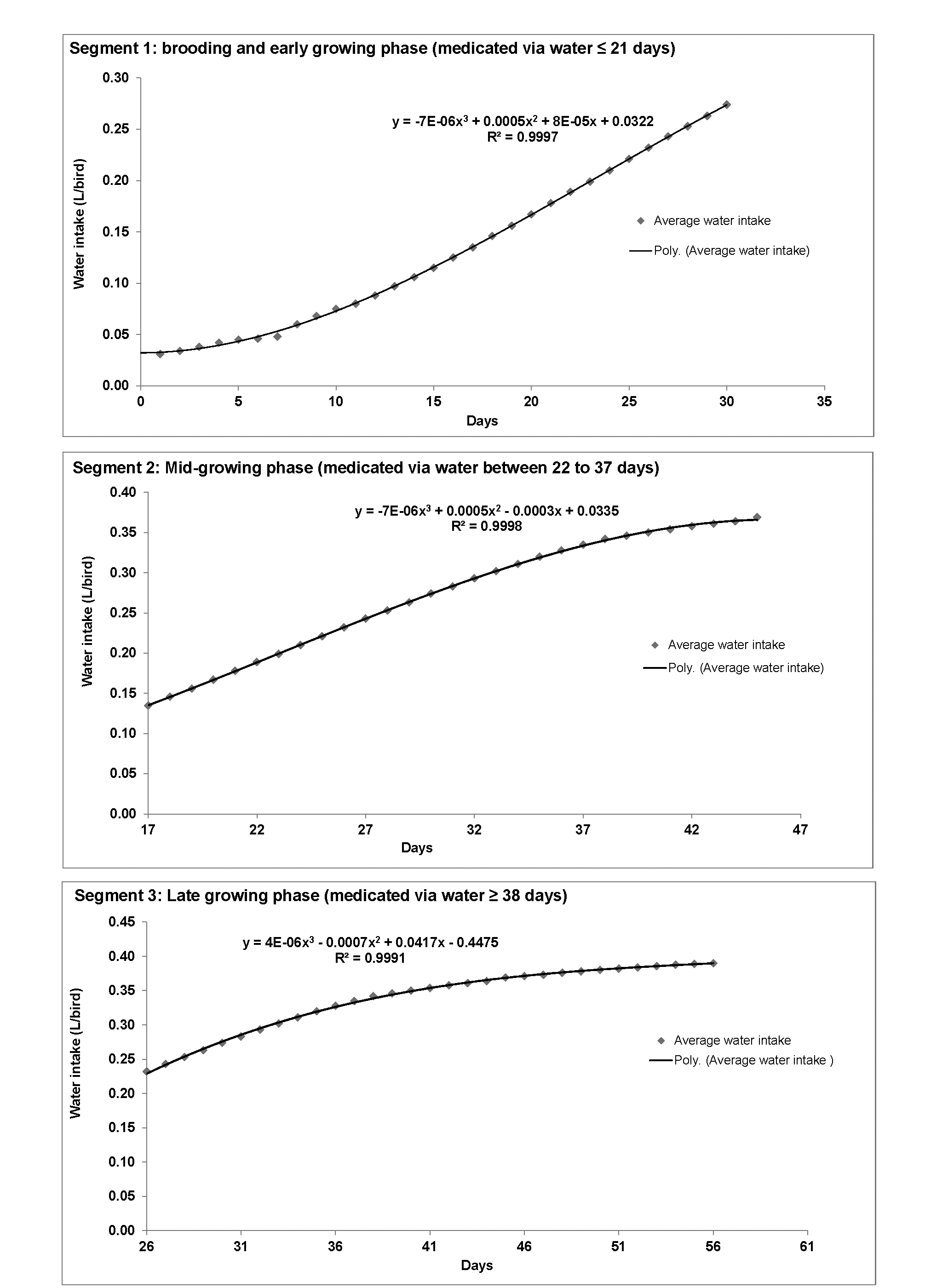

Supplement: S1 Fig — Water consumption estimates were obtained from the Nutreco Canada Inc.-Shurgain standards daily water consumption chart and a plot of intake in liters/bird/day was created (i.e., similar to the feed estimation methods). Regression parameters were calculated within Microsoft Excel by using the plotted water intake curve. As in feed, a minimum R-square value of more than 0.99 was required to be considered a good fit therefore to obtain the best fitting regression values the water consumption curve was divided into 3 segments. (TIFF) [file pone.0179384.s005.tiff]
