# Supplementary material for: Antimicrobial use surveillance in broiler chicken flocks in Canada, 2013-2015
Source: PLoS One. 2017 Jun 28;12(6):e0179384. doi: 10.1371/journal.pone.0179384 (PMC5489168; doi:10.1371/journal.pone.0179384)
Supplement: S1 Text — (DOCX) [file pone.0179384.s006.docx]

**S1 Text. Definitions and Canadian industry information used in the manuscript^[[1]](#footnote-1)^**

CIPARS – Canadian Integrated Program for Antimicrobial Resistance Surveillance. CIPARS collects, analyses, and communicates trends in antimicrobial use and in antimicrobial resistance for select bacteria from humans, animals, and retail meat across Canada (<http://www.phac-aspc.gc.ca/cipars-picra/index-eng.php>).

Flock- a group of birds hatched and delivered to a single unit (barn, floor or pen) at approximately the same day within a farm/establishment.

Farm - a broiler facility that has barns dedicated to broiler production under the supply management (Source: <http://www.chickenfarmers.ca/how-we-do-it/what-is-supply-management/>). The farm site can have multiple barns and multiple floor levels or pens within a barn. Management practices and biosecurity levels may differ from farm-to-farm.

Supply management- “is a Canadian approach to production that allows farmers across the country to match their production to Canadian demand” (Source: . <http://www.chickenfarmers.ca/how-we-do-it/what-is-supply-management/>).

Quota – “is a licence to produce and market chicken in Canada and issued by the provincial marketing boards”. For example, “in Ontario, it is issued by Chicken Farmers of Ontario (CFO) under authority delegated to it by the Ontario Farm Products Marketing Commission under the Farm Products Marketing Act”. “Quota allows a person the privilege of producing and marketing a certain number of kilograms of chicken during a specific period of time as determined by CFO. Quota represents a share of a market (Source: <https://www.ontariochicken.ca/Farmer-Member-Resources/Quota-Info.aspx>)

Quota period – an eight week production cycle in the Chicken Farmers of Canada (and respective provincial marketing board) quota allocation calendar (Source: <http://www.chickenfarmers.ca/resources/allocation-calendar-2016-2017/>).

One unit of quota - this is equivalent to 14,000 units of quota. For example, “in Ontario, as of December 2015, a unit of quota corresponds to roughly 13.0 kilograms of chicken production on an annualized basis; therefore, the 14,000 unit minimum quota would result in about 182,000 kilograms of chicken production per year (14,000 x 13.0)” (Source: <https://www.ontariochicken.ca/Farmer-Member-Resources/Quota-Info.aspx>).

1. For more information please consult the industry links. [↑](#footnote-ref-1)
